# Seasonal Pattern of Prevalence and Excretion of Eggs of *Baylisascaris transfuga* in the Brown Bear (*Ursus arctos*)

**DOI:** 10.3390/ani10122428

**Published:** 2020-12-18

**Authors:** Ladislav Molnár, Alžbeta Königová, Peter Major, Zuzana Vasilková, Martina Tomková, Marián Várady

**Affiliations:** 1 Clinic for Birds and Exotic Animals, University of Veterinary Medicine and Pharmacy, 040 01 Košice, Slovakia; ladislav.molnar@uvlf.sk (L.M.); major.dr@gmail.com (P.M.); 2 Institute of Parasitology of the Slovak Academy of Sciences, 040 01 Košice, Slovakia; vasilko@saske.sk (Z.V.); varady@saske.sk (M.V.); 3 Slovak Hydrometeorological Institute, Ďumbierska 26, 041 17 Košice, Slovakia; martina.tomkova@shmu.sk

**Keywords:** European brown bear, *Baylisascaris transfuga*, prevalence, seasonal dynamics

## Abstract

**Simple Summary:**

The main goal of this study was to monitor the seasonal dynamics of faecal egg counts (FEC) expressed as eggs per gram (EPG) and intensity of excretion of the egg nematode *Baylisascaris transfuga* in the European brown bear over three years. The number of nematode eggs in the faecal samples began to rise in the spring and peaked in the autumn throughout the study period. Presence of nematode eggs in the samples was also observed in the winter season. Of the environmental factors, the seasonal dynamics and intensity of the infection were most influenced by temperature, while humidity and mean precipitation did not affect it. Increasing mean temperatures during the winter and short or no hibernation together with the presence of *B. transfuga* infection may negatively affect the health of infected bears. Due to the zoonotic potential of the parasite and the increased occurrence of bears in the vicinity of human dwellings, the dissemination of propagative stages may also pose a threat to human health.

**Abstract:**

The seasonal dynamics of the prevalence, abundance, and mean intensity of egg excretion by the nematode parasite *Baylisascaris transfuga* in the European brown bear (*Ursus arctos*) were monitored relative to environmental factors (mean temperature, humidity, and temperature) over three years. The prevalence, abundance, and mean intensity of egg excretion tended to increase from spring to autumn throughout the monitoring period. The seasonal prevalence (84.2–90.6%), abundance (470.2–545.3 eggs per gramme (EPG) of faeces), mean intensity of excretion (558.3–602.1 EPG), and number of eggs (1150 EPG) were highest in autumn. The prevalence of eggs (up to 48.5%), abundance (37.8–60.6 EPG), and mean intensity of excretion (94.4–125.0 EPG) were high in winter, despite the period of hibernation. The seasonal dynamics of *B. transfuga* abundance in bears, the mean temperature between spring and autumn, and the seasonal trend of increase in intensity of egg excretion with temperature from winter to summer were interrelated. Abundance differed significantly between winter and autumn, spring and autumn, and summer and autumn (*p* < 0.0001) in all years and between spring and summer in 2016 (*p* < 0.005), 2017 (*p* < 0.05). *B. transfuga* abundance differed significantly between the seasons over the three years only in spring (*p* ≤ 0.0001).

## 1. Introduction

The European brown bear (*Ursus arctos*) is present in 22 European countries, with an estimated total number of approximately 17,000 individuals [1]. Most of these populations are strictly protected and are currently increasing [2]. The Carpathian population includes brown bears in Slovakia, Poland, the Ukraine, and Romania. The population in the Carpathian Mountains is estimated at about 8100 bears, and is the second largest in Europe. More than 90% of bears in Slovakia belong to the West Carpathian subpopulation, which is no longer isolated, but is again contiguous with that in the East Carpathian subpopulation in north-eastern Slovakia, south-eastern Poland, and the Ukrainian Carpathians. Some of these bears also likely have transborder home ranges [3]. Data from the State Nature Conservancy indicate that brown bears in Slovakia inhabit about 33% of the area, covering 16,000 km^2^ [4,5]. Štofik et al. [5] documented significant growth of the bear population over the last decades. Estimated population density was one bear per < 850 ha in 2001 and one bear per < 380 ha in 2014, i.e., a 2.2-fold increase in density. The estimated density in 2019 was one bear per < 310 ha [4]. The estimated population in 2014 based on faecal genetic analyses was 1256 individuals, and the current number of brown bears in Slovakia is about 1500–1600 individuals [6,7]. This bear population growth can be explained by the reduction of human settlements and increasing forestation and food sours [8], and by the protection of the brown bear. According to the Habitats Directive (Annex IV of 92/43/EEC Directive), the Slovak Act on Nature and Landscape Protection (No. 543/2002) and the Decree of the Ministry of Environment (No. 24/2003), the brown bear is a species of European importance with a high protection priority. Conservation and management are guided by the accepted management plan for large carnivores in Slovakia. The bear population has been increasing from the long-term perspective [5]. Since bears utilize food sources provided by a man for wild boar, we presume that the population growth of brown bears in region [5] can also be stimulated by supplementary feeding by hunters. This leads to sufficient food source for non-denning bears and subsequently decreased winter home range.

The changing living conditions of wild bears, their shrinking home ranges, the impact of urbanization (human density, or human agricultural land use, developed and forested) are factors supporting the transmission of infective stages of nematodes, which have the potential to cause parasitic zoonoses in humans [9]. All species of *Baylisascaris* nematodes in wild carnivores can be regarded as potentially zoonotic [10]. Kazacos [11] stated that all *Baylisascaris* species could be considered potential etiological agents of larva migrans depending on the dose of infective eggs, although *B. procyonis* and *B. columnaris* in common raccoons are the primary causative agents of zoonoses associated with wild carnivores. *B. transfuga* is a widespread pathogen in populations of free-ranging bears in many areas of the world [12,13]. Adult *B. transfuga* are first detected in bear cubs from five months of age [14], and infection decreases with age. Partridge [15] and Moran et al. [16] reported that young and immature bears were more susceptible. The pathogenicity of intestinal infection with *B. transfuga* is low in bears; one case of granulomatous peritonitis caused by nematodes was described in a cub [17]. Little information is available about the life cycle of *B. transfuga* in wild bears. The complete life cycles of most species of *Baylisascaris* are unknown [18]. Moran et al. [16] stated that infection with *B. transfuga* could be direct or via a paratenic host, such as wild rodents. Encapsulated larvae of *B. transfuga* have been detected in the mesentery and the wall of the large intestine of the grey-sided vole (*Myodes rufocanus*) and the tundra vole (*M. oeconomus*) [19]. Abdelrasoul and Fowler [20] found that the life cycle was direct, but could also be indirect via encysted larvae using various rodents, birds, or insects as intermediate hosts. Transmammary and transplacental transmission are apparently unlikely [11]. In contrast, larvae migrate through various tissues in rodents, e.g., white mice and Mongolian gerbils, which are susceptible to experimental infection with *B. transfuga*, growing and developing to the third stage and causing various degrees of visceral, neural, or ocular larva migrans [21]. Larva migrans are the most serious complication associated with *Baylisascaris*, mainly caused by larvae of the common raccoon parasite *B. procyonis* in intermediate and occasional hosts. *Baylisascaris procyonis* is the most common clinical larva migrans in animals, in which it is usually associated with fatal or severe neurological disease. It is known for aggressive somatic migration, larval invasion of the central nervous system, and the capability for continued larval growth within intermediate hosts [22]. A single larva in the brain of a rodent is enough to cause clinical signs, even death [11,23]. In infants and young children, in its most severe form, *B. procyonis* is a rare cause of fatal or neurologically devastating neural larva migrans [22].

Definitive hosts of *B. transfuga* rarely show clinical signs unless heavy infections of adult worms block the digestive tract. Wallach and Boever [24] reported symptoms of diarrhoea, anorexia, and dry and severe cough, with the occasional presence of parasites in the faeces. Heavy infestation can lead to intestinal obstruction and subsequently to death [24,25]. Foster et al. [14] reported that black bear cubs harboured 1–39 nematodes in their intestines. Infected captive bears may shed as many as 10,000–20,000 eggs per gramme (EPG) of faeces [26] and thereby heavily contaminate their domestic areas.

Most information about the prevalence of *B. transfuga* in populations of wild bears has been from the American, Canadian, and Russian populations of black, brown, grizzly, and polar bears, respectively [14,27,28,29,30,31,32,33]. Information about the prevalence of *B. transfuga* in populations of wild brown bears in Europe is limited [1,34,35,36,37,38,39], and little is known about the seasonal dynamics of *B. transfuga* in these populations [36,37,38,40]. The seasonal dynamics of *B. transfuga* in wild carnivores can also be influenced by external factors such as temperature, humidity, and rainfall. Sufficient humidity and optimal temperature support the growth and survival of infective stages of nematodes, leading to higher contamination of carnivore environments. Climatic factors can impact the growth and survival of infective stages of *Baylisascaris* nematode. Temperature, humidity, and rainfall can affect environmental moisture retention of soils that were associated with transmission to paratenic hosts by improving viability at or near the surface [9]. Moisture and particle size also can affect egg viability, keeping viable eggs and can increase the potential risk of infection [41].

The present study determined the seasonal changes during a three-year period in the prevalence of *B. transfuga* eggs in faecal samples of the brown bear in Poloniny National Park, Eastern Carpathians, in Slovakia.

## 2. Materials and Methods

Permission for conducting a study was granted by the Administration of the Poloniny National Park. According to national law, formal ethical approval is not required for studies based on the monitoring of indirect signs of animals, such as collection of faecal samples.

### 2.1. Study Area

The study was conducted at the East Carpathians Biosphere Reserve (ECBR, UNESCO Man and Biosphere), the first European trilateral Man and Biosphere reserve (Slovakia/Poland/Ukraine) and the second largest biosphere reserve in Europe. The reserve covers an area of 208,089 ha (the Polish, Slovak, and Ukrainian parts occupy 52.2%, 19.59%, and 28.16%, respectively) includes six large protected areas, and contains the largest European complexes of original beech forests, and East Carpathian montane meadows. Poloniny National Park, part of ECBR, is the largest forest complex in Slovakia, with an area of 29,805 ha (a buffer zone of 10,973 ha). Endemic communities are predominant, with a large local population of European brown bears. The estimated density varies between 5 and 11 bears per 100 km^2^.

### 2.2. Parasitological Examinations

**Figure 1:**
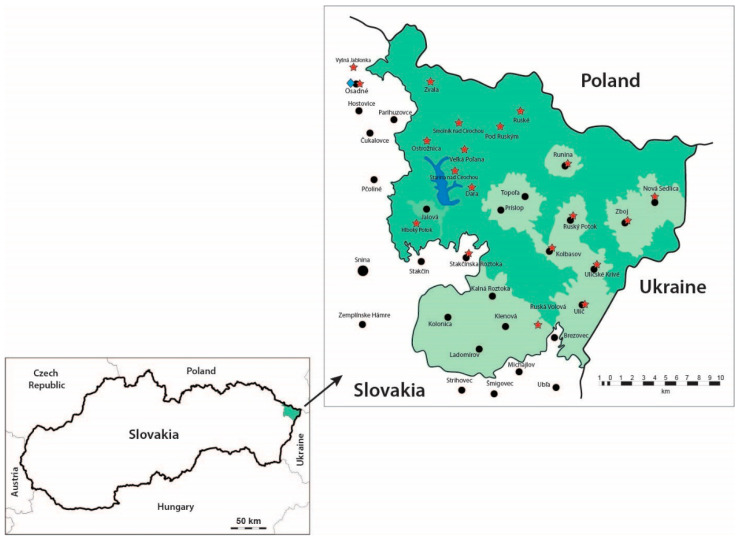
Distribution of the brown bear faecal samples in the study area in Poloniny National Park, Slovakia (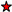
—sampling sites; 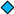
—Hydrometeorological station, National Park Poloniny in Osadné).

A total of 524 faecal samples from brown bears were collected at the Poloniny National Park in north-eastern Slovakia from January 2016 to December 2018. The samples were collected with the assistance of the staff of the national park from several bear territories with presumed or reported occurrences of the animals (Figure 1). The national management plan for large carnivores estimates that the population size consists of around 35 bear individuals in the present time (2016–2018). Collection of bear faeces was roughly in the same point of an area usually correlated with hunters’ feeding places, salt lick sites, or snow tracking in the winter period. During the snow season, bear tracks were followed in opposite direction of the bear movement to avoid disturbing the animal. Length and width of the footprint was recorded, if possible, on the snow or mud to identify individual bears. In the periods without snow cover, the faeces were collected systematically during the whole period following the method of Persson [42] et al. (2001) from the areas with reported and suspected occurrence of bears (orchards, roads, feeding racks). A GPS device (Garmin eTrex Vista H) was used to store the position and altitude of exact localities to avoid multiple sampling from the same scats. The numbers of faecal sampled was determined monthly.

Each faecal sample was stored in a plastic container at 4 °C and subsequently frozen until further processing. Frozen samples were thawed 24 h prior to coprological examination. All samples were analysed no later than two weeks after collection. The faecal egg count (FEC) was used to monitor the prevalence of the nematode *B. transfuga* in brown bears. Baylisascaris FEC were performed using the coprological McMaster technique according Coles et al. [43] with Sheather’s sugar flotation solution (1.25 specific gravity) and expressed as EPG (eggs per gram). Identification of *Baylisascaris transfuga* eggs was performed based on the morphology of the eggs by Sprent [44], and Kazacos and Turek [45]. The data on *B. transfuga* eggs were used to estimate prevalence, based the recommendations of Margolis et al. [46]: Prevalence (%), number of bears infected with *B. transfuga* eggs excreted in faecal samples divided by total number of bears examined. Mean excretion of eggs (EPG), total number of excreting of *B. transfuga* eggs in a bear faecal sample divided by number of infected bears; and abundance (EPG), total number of excreting of *B. transfuga* eggs in a bear faecal sample divided by total number of bears examined.

Basic climatological data, mean air temperature, minimum and maximum air temperatures, mean rainfall, and mean relative air humidity for 2016–2018 were obtained from the Slovak Hydrometeorological Institute (SHI), from the particular Hydrometeorological station, National Park Poloniny in Osadné (Figure 1). The data were analysed for the four seasons to assess the influence of meteorological factors: Spring (March–May), summer (June–August), autumn (September–November), and winter (December–February). The Kruskal–Wallis test (nonparametric ANOVA) was used to identify significant differences between seasonal prevalences (spring, summer, autumn, and winter) at *p* < 0.05 (GraphPad Software, San Diego, CA, USA).

## 3. Results

### 3.1. Seasonal Prevalence of B. transfuga in the European Brown Bear

**Figure 2:**
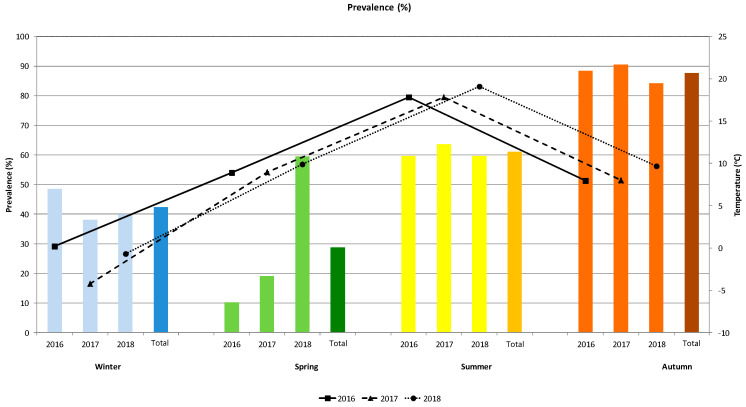
Prevalence of eggs of the nematode *B. transfuga* in the faecal samples of the brown bear (*Ursus arctos*) and mean temperature (°C) during different seasons of the three-year survey.

The seasonal dynamics of the prevalence of *B. transfuga* eggs in brown bears (a total of 524 faecal samples) in connection to temperature for three years is shown in Figure 2. The prevalence of baylisascariasis steadily increased over the years from spring to autumn. The difference of prevalence in spring is not statistically significant between 2016 (10.3%) and 2017 (19.1%), but significantly higher prevalence was observed in 2018 (59.5%). Prevalence in summer was similar (59.6–63.6%). Prevalence was highest in autumn (from 84.2 to 90.6%). Prevalence in winter was high (38.1–48.5%) in non-denning bears. Similar patterns of variation were observed between the dynamics and variations in mean temperature was noted in spring and autumn (temperature was recorded in the same range and no interannual differences among temperature a same season were determined) (Figure 2).

### 3.2. Seasonal Abundance of B. transfuga in the European Brown Bear

**Figure 3:**
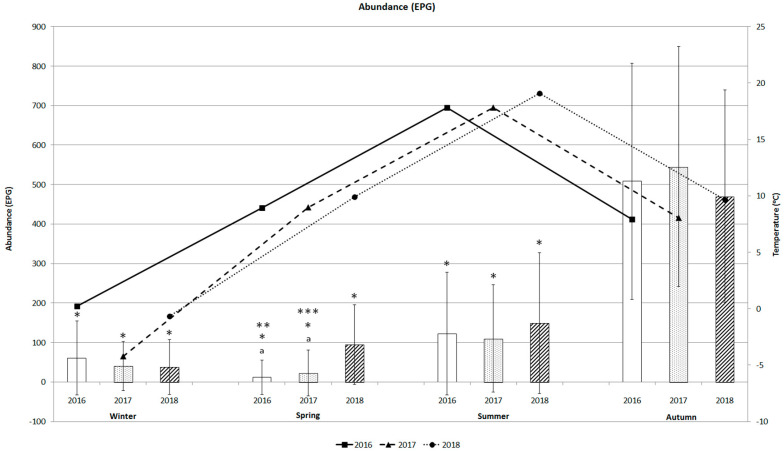
Abundance (mean EPG) of *B. transfuga* eggs in the faecal samples of the brown bear (*Ursus arctos*) and mean temperature (°C) during different seasons of the three-year survey; a significant difference between spring 2016 and 2017, and spring 2018, *p* < 0.0001; *, significant differences between autumn and winter, spring, and summer 2016, 2017, and 2018; *p* < 0.0001; **, significant difference between spring and summer 2016, *p* < 0.005; ***, significant difference between spring and summer 2017, *p* < 0.05.

Figure 3 shows the seasonal dynamics of the abundance of baylisascariasis in bear faecal samples and the variations in mean temperature during the three-year period, with an increasing trend from spring to autumn. Abundance was lowest in spring (11.5 ± 43.6 EPG ± SD), with a maximum EPG of 250 (Table 1). Abundance in summer varied from 110.0 ± 135.9 to 149.0 ± 178.6 EPG during the study period. Although abundance was higher in winter (37.8 ± 70.0 to 60.6 ± 93.3 EPG) than in spring, abundance was not so high in the other season. The similar patterns of variation between the abundance of *B. transfuga* and variations in mean temperature were recorded from winter to summer. The abundance of baylisascariasis increased with temperature from winter to summer for the three years, except in spring 2016 and 2017, when EPGs were lower (11.5 ± 43.6 and 22.6 ± 57.6). Abundance increased rapidly in autumn (470.2 ± 268.9 to 545.3 ± 304.2 EPG) (Figure 3), similar to the autumn prevalence rates (84.2–90.6%) (Figure 2).

Seasonal *B. transfuga* eggs’ abundance in brown bears differed significantly only between spring 2016 and 2018 (*p* < 0.0001), and spring 2017 and 2018 (*p* = 0.0001). *B. transfuga* eggs’ abundance in brown bears differed significantly between winter and autumn (*p* < 0.0001), spring and autumn (*p* < 0.0001), summer and autumn (*p* < 0.0001), and spring and summer 2016–2017 (*p* < 0.005; *p* < 0.05) (Figure 3).

### 3.3. Seasonal Mean Excretion of B. transfuga Eggs in the European Brown Bear

**Figure 4:**
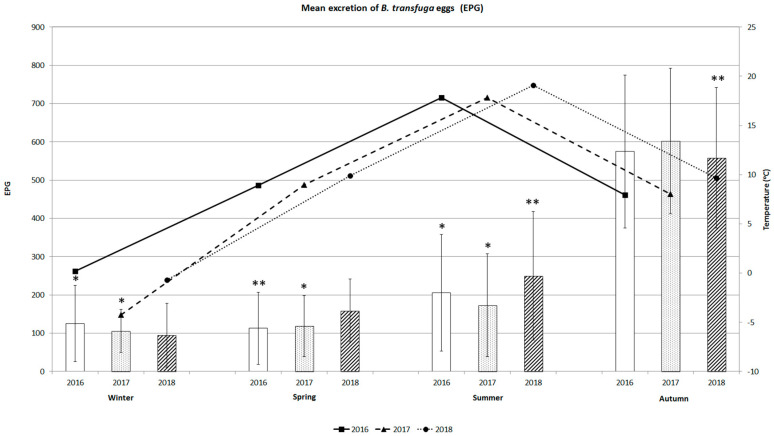
Mean excretion of *B. transfuga* eggs (EPG) in the brown bear (*Ursus arctos*) and mean temperature (°C) during different seasons of the three-year survey; *, significant differences between autumn, winter, and summer 2016 and between winter, spring, and summer 2017; *p* < 0.0001; **, significant differences between spring and autumn 2016 and between winter, summer, and autumn 2018, *p* < 0.00.

The seasonal dynamics of mean excretion of *B. transfuga* eggs in the faecal samples from brown bears and the variations in mean temperature during the three years are shown in Figure 4. Mean excretion of *B. transfuga* eggs in bears seasonally increased from winter to autumn in all three years. The excretion of eggs in 2016 differed significantly between winter and autumn (*p* < 0.0001), spring and autumn (*p* < 0.005), and summer and autumn (*p* < 0.0001). The excretion of eggs in 2017 differed significantly between winter and autumn, spring and autumn, and summer and autumn (*p* < 0.0001 in all cases). Mean excretion did not differ significantly between the seasons over the three years. The intensity of excretion in 2018 differed significantly between winter and summer and winter and autumn (*p* < 0.005 in both cases). Mean excretion was lowest in winter (94.4 ± 83.8 to 125.0 ± 100.0 EPG ± SD), with a maximum of 400 EPG (winter 2018). Mean excretion was low in spring 2016 (112.5 ± 94.7 EPG), with a maximum of 250 EPG. Mean excretion increased in summer, with EPGs varying between 172.9 ± 134.7 and 205.4 ± 152.4 EPG, with a maximum of 550 EPG. Mean excretion was highest in autumn (from 558.3 ± 189.4 to 602.1 ± 259.7 EPG), with a maximum of 1550 EPG (2017).

The excretion of parasitic eggs was highest in autumn, with the maximum EPG over the three years. Mean excretion tended to increase with temperature from winter to summer.

Any relationship was not observed between data on precipitation and humidity and seasonal results of FEC. In our study, we also investigated the relationship between the occurrence of *B. transfuga* and mean precipitation and humidity (Table 2). We did not observe any relationship between our findings and any of the mentioned climatic parameters.

## 4. Discussion

Our study describes seasonal patterns of mean excretion, abundance, and prevalence of *B. transfuga* eggs by brown bears in a Central Europe area. The prevalence, intensity, and geographic distribution of *B. transfuga* infections have been confirmed worldwide in free-ranging bears such as the American black bear and grizzly bear, the European brown bear, and the polar bear. The seasonal dynamics of *B. transfuga* infections in populations of wild bears could also be influenced by the large differences between bear species and the genetic variability and adaptability of nematodes to a wide range of external temperature differences. Further studies would be useful to clarify the intra- and interspecific relationships amongst *B. transfuga* from different geographic areas and bear species and to address epidemiological and zoonotic patterns of this bear nematode.

Only limited data has been reported for the seasonal dynamics of *B. transfuga* prevalence in populations of free-ranging bears, with some conflicting results. The prevalence of *B. transfuga* in American and Canadian populations of wild bears has been reported in several studies [14,25,28,29,30,32,47,48,49,50], but only a few studies have attempted to investigate seasonal trends in prevalence. One of the studies comparing the incidence of eggs confirmed a higher prevalence in spring (42%) compared to autumn prior to denning (13%) in black bears in Canada [29]. Such an opposite trend to our findings may have been due to the development of larvae surviving hibernation [29]. Several studies have provided evidence that intestinal parasites that feed directly on the host organism pass from the gastrointestinal tract before the onset of hibernation [28,49]. Choquette et al. [49] and Rausch [47] also hypothesised that nematodes were eliminated prior to hibernation in bears. Rogers [28] found that bears excreted *B. transfuga* eggs in the spring soon after hibernation and found evidence of egg excretion immediately prior to denning, so whether or not parasitic infections are cleared during winter hibernation remains unclear, and the apparent elimination of *B. transfuga* during hibernation is poorly understood. Rogers [28], however, confirmed that baylisascariasis in a population of wild bears occurred mainly in summer (75%). Similarly, Manville [50] reported the highest prevalence of *B. transfuga* eggs (64%) and adult parasites (89%) of American black bears (*Ursus americanus*) in summer based on faecal floatation and necropsy. Additionally, Clark et al. [48] found that the incidence of *B. transfuga* infection peaked from June to August and was lower during autumn and winter. Gau et al. [30] and Catalano et al. [32], however, observed the opposite seasonal trend, with peaks in autumn in black bears and grizzly bears in Canada, consistent with our results. Gau et al. [30] reported that the prevalence of gastrointestinal parasites, including *Baylisascaris*, in grizzly bear faeces was low in spring (31%) and high in autumn (58%) based on faecal floatation. Such dynamics may have been due to the expulsion of adult nematodes before hibernation and subsequent reinfection in spring, because bears restrict their food intake and evacuate their bowels shortly before hibernation. Catalano et al. [32] observed the seasonal trend in prevalence based on the collection of nematodes at necropsy, with peaks in autumn (87.5%) in grizzly and black bears.

Little information is available on the prevalence of *B. transfuga* in populations of wild brown bears in Europe [1,35,36,37,38,39,40]. Only a few of these studies have attempted to evaluate the seasonal dynamics of this nematode. De Ambrogi et al. [39] described *B. transfuga* prevalence (13.5%) in free-ranging European brown bears in Croatia based on a coprological study. Goldova et al. [36] identified gastrointestinal parasites in faecal samples in Slovakia, with a predominant prevalence of *B. transfuga* (72.34%). The prevalence of baylisascariasis defined by FEC coprological method in brown bears during the three years of our study was highest in autumn. The FEC coprological non-invasive method is routinely used for detecting *Baylisascaris* in raccoon faeces and in the environment [51,52]. Limitation of this coprological non-invasive method could be false-negative stool examination results [51,52]. Despite the large number of eggs that are eliminated, it is recommended that three daily faecal samples be examined for *Baylisascaris* in raccoons [51], but it was not possible in wild bears. The highest prevalence in autumn in our study may be highly influenced by interactions such as age and sex of the examined bears. The intensity of egg excretion at one sampling site may be influenced by the fact that the source of the samples may be individuals of different ages. Among other factors, the age composition of the studied bears may have had a significant effect on the high prevalence of *B. transfuga* in our results. As is well known, juvenile raccoons are susceptible to infection through ingestion of eggs, while the older bears become infected through paratenic hosts [11], and immunity to larvae is developed following primary exposure in young animals, which decreases the burdens of parasites in future infections [51]. Young racoons can produce an egg output of 115,000–179,000 eggs per day [53]. Differences in prevalence between the sexes were also confirmed in raccoons, where higher prevalence was recorded in males than in females. Transplacental and transmammary transmission has been described in raccoon females [11,54]. Previous ingestion of larvated eggs is probably cumulative and may be affected by factors such as prepatent period (a prepatent period of 7–10 weeks), and also bear physiology changes as hyperphagia before hibernation.

The seasonal dynamics of *B. transfuga* prevalence in bears could be more influenced by internal factors such as hormonal and metabolic changes occurring before, during, and after bear hibernation. The occurrence of roundworms in hibernating bears in spring could be related to brown fat and the hypothalamic hormone, which affect the bear’s carbohydrate metabolism by up to 75%. Bear hibernation could affect the metabolism of intestinal nematodes. Glycogen is apparently synthesized from hexoses and plays an important role in supplying energy to nematodes [37]. Hibernation in relation to parasite metabolism has been described by several authors [28,49]. Finnegan [37] hypothesised that adult parasites usually died during hibernation in winter due to carbohydrate deficiency and subsequently disintegrated. Bears accumulate large stores of body fat during late summer and autumn necessary for winter hibernation. They have a hyperphagic phase in which food intake increases 2–3-fold, and their body weight can increase by 30–35% [55]. Hyperphagia provides a sufficient source of carbohydrates for the survival and reproduction of nematodes [37]. Adult nematodes accumulate in the intestinal tracts of bears beginning in spring, which could be correlated with the highest seasonal peak in the prevalence of *B. transfuga* in bears in autumn. However, the mechanism of hibernation in bears is not well known, but is likely to be influenced by the interaction of several stimuli, including reduced food availability, low temperature, snowfall, daylight, physical conditions, sex, age, and reproductive status, and may also be influenced by bear species [56].

The high seasonal prevalence of *B. transfuga* in non-denning bears in winter is a concern and may be a consequence of the increase in mean temperatures due to global warming or to supplemental food sources provided by hunters in winter. Insufficient hibernation time due to warm winters and the consequent high prevalence of *Baylisascaris* infection can negatively affect the health of infected bears. The bear’s carbohydrate metabolism in non-denning bears in winter is still built up. Is it assumed that bear hibernation could be negatively affected by the metabolism of intestinal nematodes and lead to “clearing up”. This hypothesis can mainly affect juvenile subadults in bears which did not build up sufficient fat deposits and contribute to worsening body condition in spring time. Hibernation in relation to parasite metabolism has been described by several authors [28,49]. Bears, however, may not hibernate in mild winters [57]. The immobilisation of bears could be associated with mild winter temperatures due to global warming. The availability of sufficient food sources until late autumn and winter is another important factor associated with the delay or elimination of bear hibernation. Additionally, intensive feeding of wild bears with maize, as a source of carbohydrates, led to the activity of bears during winter [58,59].

The prevalence of baylisascariasis in brown bears during the three years of our study was highest in autumn. Our findings are consistent with most studies that have investigated the seasonal dynamics of *B. transfuga* in bears [38,40]. These studies found that the prevalence was highest in autumn or winter and lowest in spring. Orosová et al. [40], however, found that the prevalence of *Baylisascaris* was highest in autumn (85.71%) and early winter (100%). Similar results were reported by Major et al. [38] in their coprological study, who also observed that the prevalence of *B. transfuga* was highest in autumn and winter and lowest in spring, which corresponds to the results of our study. Several studies have confirmed the occurrence of parasites in bears during the winter, when bears should hibernate [36,38,40].

We also observed a seasonal occurrence of nematode eggs in bear faeces during the winter. Our study recorded the prevalence of *B. transfuga* excreted eggs in faecal samples in the bears up to 48.5%. Štrkolcová et al. [34] suggested that the high prevalence of endoparasites in winter was associated with relatively warm winters, which shortened or eliminated hibernation. However, it has been reported that seasonal pattern of *B. procyonis* in racoons may be disturbed in warmer southern regions of the United States [60,61]. So, the goal of our study was to confirm or refute this temporal seasonal pattern in the conditions of mild climate in Central Europe. The increase in temperatures during the winter has been observed in the mild climatic conditions of Central Europe in recent years. For this reason, we decided to monitor the prevalence of *B. transfuga* and the nematode egg excretion in brown bears’ faecal samples during all seasons at monthly intervals over 3 years. To a lesser extent, soil type, habitat fragmentation, and other environmental components, such as road density, have been assessed as risk factors for parasitic infection [9]. Increased risk of transmitting *Baylisascaris* infection to paratenic hosts may affect the factors such as moisture retention and size of particles of the soil. Moisture and particle size also can affect egg viability [41]. For this reason, we decided to compare climatic parameters (temperature, humidity, rainfall) with the nematode egg excretion in brown bears in the same period. Environmental factors are related to the secretion of eggs in the same period of time, but mainly affect the accumulation of infectious stages in previous periods. Additionally, since eggs can remain present and viable for many years following deposition [62], seasonal or annual trends may be masked. However, our results confirmed the temporal pattern with a peak FEC in the autumn and a subsequent winter decline without proving the influence of climatic factors.

Our results did not confirm the effect of temperature, precipitation, and humidity on excretion of *Baylisascaris transfuga* eggs in brown bears in the mild climate of Central Europe. Prevalence, abundance, and mean egg excretion may be highly influenced by interactions such as age, sex, hyperphagia before hibernation, and previous larvated eggs ingestion, which are most probably cumulative during the prepatent period.

*B. transfuga* was not studied in wild brown bears in Europe as *B. procyoonis* in racoons in America. In general, information about the prevalence of *B. transfuga* in wild European bears is limited, and there is no data on environmental effects on the seasonal dynamics of *B. transfuga* in wild bears in Europe.

A national brown bear management plan has yet to be established, despite repeated calls by the Standing Committee of the Bern Convention and the Council of Europe. There has been little meaningful cooperation with neighbouring states sharing the same bear population (Czech Republic, Poland, Ukraine) and so consideration of management at population level, also emphasised by international experts, has been inadequate. Loss, fragmentation, and disturbance of habitat are potentially major threats to wildlife in the Carpathian Mountains. Adequate steps need to be taken to prevent permanent isolation of habitat and wildlife, including bears [63]. Research on ecology is very important to allow better understanding of the needs of bears as well as the causes of bear–human conflicts. Habitat use, social organisation, and dispersal are especially pertinent. Studies focused on the ecology and management of the brown bear in Central/Eastern Europe are needed in the future.

## 5. Conclusions

The prevalence, abundance, and mean excretion of *B. transfuga* eggs in bears were highest in autumn in all three years. We observed a relationship between the dynamics of *B. transfuga* prevalence in bears and changes in mean temperature in spring and autumn, and a seasonal trend of increasing intensity of parasitic infection from spring to autumn. The increase in the bear population, shrinking territories, and the increase in occurrence in urban areas, however, are also alarming. The dissemination of the propagative stages of bear nematodes in both urban and rural ecosystems near human dwellings could pose a risk to human health.

## Figures and Tables

**Table 1:** Number of examined and infected faecal samples, minimum and maximum eggs per gram (EPG) of faeces, abundance, mean intensity of excretion, and prevalence of *B. transfuga* eggs in the brown bear (*Ursus arctos*) during different seasons of the three-year survey.

Season	Year	Number of Examined	Number of Infected	Minimum EPG	Maximum EPG	Abundance (EPG ± SD)	Mean Intensity of Excretion (EPG ± SD)	Prevalence (%) 95% CI
Winter	2016	33	16	0	350	60.6 ± 93.3	125.0 ± 100.0	48.5 (0.308–0.664)
	2017	21	8	0	200	40.5 ± 62.5	106.3 ± 56.3	38.1 (0.181–0.616)
	2018	45	18	0	400	37.8 ± 70.0	94.4 ± 83.8	40.0 (0.257–0.557)
Spring	2016	39	4	0	250	11.5 ± 43.6	112.5 ± 94.7	10.3 (0.029–0.242)
	2017	42	8	0	250	22.6 ± 57.6	118.8 ± 79.9	19.0 (0.086–0.341)
	2018	37	22	0	350	94.6 ± 101.2	159.1 ± 82.6	59.5 (0.421–0.753)
Summer	2016	47	28	0	500	122.3 ± 154.9	205.4 ± 152.4	59.6 (0.443–0.736)
	2017	55	35	0	500	110.0 ± 135.9	172.9 ± 134.7	63.6 (0.496–0.762)
	2018	52	31	0	550	149.0 ± 178.6	250 ± 167.8	59.6 (0.451–0.73)
Autumn	2016	43	38	0	1300	508.1 ± 299	575.0 ± 249.0	88.4 (0.749–0.961)
	2017	53	48	0	1550	545.3 ± 304.2	602.1 ± 259.7	90.6 (0.793–0.968)
	2018	57	48	0	950	470.2 ± 268.9	558.3 ± 189.4	84.2 (0.721–0.925)

**Table 2:** Basic climatological parameters, mean temperature, minimum and maximum temperatures, mean rainfall, and mean humidity, during the three-year survey (2016–2018).

Season	Temperature (°C)									Mean Rainfall (mm)			Mean Humidity (%)		
	2016			2017			2018			2016	2017	2018	2016	2017	2018
	Mean	Min	Max	Mean	Min	Max	Mean	Min	Max						
Winter	0.2	−16.6	14.5	−4.2	−20.5	12.5	−0.7	−14.2	10.5	90.8	56.3	66.8	83.1	85.5	83.2
Spring	8.9	−6.2	26.9	9.0	−3.4	26.3	9.9	−18.0	27.6	53.9	62.1	70.9	76.9	74.2	73.7
Summer	17.8	3.1	32.2	17.8	4.2	32.0	19.1	4.7	30.7	118.4	88.1	105.6	72.9	75.6	77.8
Autumn	7.9	−8.9	28.0	8.0	−7.2	28.5	9.7	−13.0	28.6	87.7	99.8	40.3	82.8	85.5	79.7

Source: SHI, Bratislava, Hydrometeorological station Osadné, SR.
